# Tetrandrine identified in a small molecule screen to activate mesenchymal stem cells for enhanced immunomodulation

**DOI:** 10.1038/srep30263

**Published:** 2016-07-26

**Authors:** Zijiang Yang, John Concannon, Kelvin S. Ng, Kathleen Seyb, Luke J. Mortensen, Sudhir Ranganath, Fangqi Gu, Oren Levy, Zhixiang Tong, Keir Martyn, Weian Zhao, Charles P. Lin, Marcie A. Glicksman, Jeffrey M. Karp

**Affiliations:** 1 Harvard-MIT Health Sciences and Technology, Cambridge, MA, US; 2 Department of Medicine, Division of Biomedical Engineering, Brigham and Women’s Hospital, Harvard Medical School, Boston, MA, US; 3 Harvard Stem Cell Institute, Cambridge, MA, US; 4 Wellman Center for Photomedicine, Massachusetts General Hospital, Boston, MA, US; 5 Advanced Industrial Technology Research Institute, Shanghai Jiao Tong University, Shanghai, China; 6 Laboratory for Drug Discovery in Neurodegeneration, Harvard NeuroDiscovery Center, Brigham and Women’s Hospital, Harvard Medical School, Cambridge, MA, US; 7 Regenerative Bioscience Center, Department of Animal and Dairy Science, and College of Engineering, University of Georgia, Athens, GA, US; 8 Department of Chemical Engineering, Siddaganga Institute of Technology, Tumkur, India; 9 Department of Pharmaceutical Sciences, Sue and Bill Gross Stem Cell Research Center and Chao Family Comprehensive Cancer Center, Department of Biomedical Engineering, and Edwards Lifesciences Center for Advanced Cardiovascular Technology, University of California, Irvine, CA, US.

## Abstract

Pre-treatment or priming of mesenchymal stem cells (MSC) prior to transplantation can significantly augment the immunosuppressive effect of MSC-based therapies. In this study, we screened a library of 1402 FDA-approved bioactive compounds to prime MSC. We identified tetrandrine as a potential hit that activates the secretion of prostaglandin E2 (PGE2), a potent immunosuppressive agent, by MSC. Tetrandrine increased MSC PGE2 secretion through the NF-κB/COX-2 signaling pathway. When co-cultured with mouse macrophages (RAW264.7), tetrandrine-primed MSC attenuated the level of TNF-α secreted by RAW264.7. Furthermore, systemic transplantation of primed MSC into a mouse ear skin inflammation model significantly reduced the level of TNF-α in the inflamed ear, compared to unprimed cells. Screening of small molecules to pre-condition cells prior to transplantation represents a promising strategy to boost the therapeutic potential of cell therapy.

Mesenchymal stem cells (MSC) represent a promising cell type for therapeutic immunomodulation and tissue regeneration. Transplanted MSC can exert their therapeutic effects through several pathways including differentiation into mature cell types; mitochondrial transfer; secretion of regulatory and trophic factors (secretome) in response to biological stimuli; or through release of extracellular vesicles carrying mRNA or miRNA^1^,^2^,^3^,^4^,^5^. Importantly, MSC exhibit a robust immunomodulatory effect^6^,^7^,^8^. Although the underlying mechanisms have yet to be conclusively elucidated, MSC have been shown to modulate the function of cell populations including T and B lymphocytes^9^,^10^,^11^, natural killer cells^12^,^13^, and antigen-presenting cells such as dendritic cells and macrophages^7^,^14^,^15^,^16^. While most studies suggest that MSC can function through an immunosuppressive/inhibitory role, others show that MSC can exhibit pro-inflammatory properties^17^,^18^. Due to the high heterogeneity of MSC between donors, tissue origins and culture methods, the profiles of secretome produced by MSC are highly variable. Thus, manipulation of MSC prior to transplantation is important to consider to maximize immunomodulatory potency and control therapeutic outcomes. In an attempt to increase the immunomodulatory potency of MSC, several strategies have been examined. IFN-γ priming of MSC has been explored to enhance direct and indirect inhibitory modulation of T cell responses^19^ by inducing immunosuppressive factors such as indoleamine-2,3-dioxygenase (IDO), inducible nitric oxide synthase (iNOS), and prostaglandin E2 (PGE2). The clinical efficacy of IFN-γ-primed MSC as compared to unprimed MSC was reviewed in detail by Sivanathan, *et al*.^19^. MSC genetically transfected with interleukin-10 (IL-10) mRNA showed a superior anti-inflammatory effect *in vivo*^20^. Bartosh, *et al*. reported that MSC spheroids formed in hanging drops express high amount of TNF-α stimulated gene/protein 6 (TSG-6), stanniocalcin-1, IL-24, TNF-α-related apoptosis inducing ligand, and CD82^21^. MSC engineered with budesonide-loaded PLGA microparticles have also been explored to enhance IDO activity^22^. In particular, small molecule pre-conditioning represents an attractive approach that is amenable to treatment of large numbers of cells for clinical scale production, yet to date, most studies modified MSC function by utilizing limited selection of well-established factors to manipulate classic immunoregulatory genes. In this study, we established a high-throughput screening (HTS) protocol to identify bioactive molecules that can specifically enhance secretion of a candidate immunomodulatory molecule, Prostaglandin E2 (PGE2) that has been implicated as a key component of the MSC secretome^8^,^23^,^24^,^25^,^26^.

## Results

### Development of high-throughput screen and selection of a positive control

**Figure 1:**
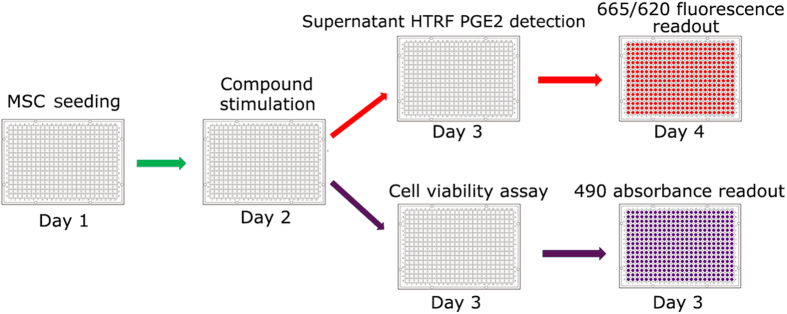
A schematic outline of the HTS approach. Cells were seeded into a 384-well plate on Day 1, and compounds were added on Day 2. On Day 3, the supernatant from each well was collected. The cells were re-incubated in fresh medium containing MTS reagent for 2 h to assess cell metabolic activity (purple path), while the collected supernatants were assayed for another 24 h (Day 4) for (red path) HTRF-based PGE2 detection.

To develop an assay to detect compounds that enhance PGE2 secretion of MSC, we implemented a strategy using primary human MSC from healthy donors. We titrated the cell number, examined the time course, and identified the best media for the screen. We found that incubating 1500 cells/well in 384-well format overnight and assaying 24 h later was best for cell metabolic activity and for measuring secreted PGE2 by Homogeneous Time-Resolved Fluorescence (HTRF) technology. Figure 1 illustrates the outline of the screen. TNF-α and IFN-γ were both reported to activate PGE2 secretion^27^,^28^,^29^. To select a suitable stimulus as a positive control, we treated MSC with 100 ng/mL TNF-α or 100 ng/mL IFN-γ and assayed for PGE2 after 6, 12, and 24 h. TNF-α induced PGE2 secretion as early as 6 h and the amount of PGE2 appeared to increase linearly throughout the 24 h assay (Supp. Fig. 1A). Neither treatment affected cell proliferation or metabolic activity (Supp. Fig. 1B). Therefore we chose to treat MSC with 100 ng/mL TNF-α for 24 h as the positive control.

### Comparison between serum-containing αMEM and STEMPRO^®^ MSC SFM

MSC had a smaller, more spindle-shaped morphology in STEMPRO^®^ MSC SFM than in serum-containing αMEM (Supp. Fig. 2A). PGE2 secretion induced by TNF-α was relatively low (<20%) when MSC were cultured in serum-containing αMEM for 24 h, but increased to ~50% in STEMPRO^®^ MSC SFM (Supp. Fig. 2B). We therefore chose to use STEMPRO^®^ MSC SFM to culture MSC to maximize the sensitivity of detection.

### Assessment of DMSO concentration

To establish the HTS protocol, we tested whether the addition of DMSO (as a standard solvent for our library compounds) would affect the stability of PGE2 in the supernatant, the metabolic activity of MSC, or the activation by TNF-α. A 24-hour incubation of 250 pg/mL recombinant PGE2 into media containing 0%, 0.5% or 2% DMSO did not reduce the ability of HTRF to quantify levels of PGE2 (Supp. Fig. 3A), suggesting that DMSO affected neither detection nor stability of PGE2. Next, we tested the metabolic activity of MSC in media containing 0.5% or 2% DMSO after a 24-hour incubation using the MTS assay and found no effect on the number of viable cells (Supp. Fig. 3B). The secretion of PGE2 by MSC induced by TNF-α was not affected by treatment with 0.5% DMSO, but decreased by more than 50% in 2% DMSO (Supp. Fig. 3C). Since the final concentration of DMSO in the compound library screen was less than 0.1%, we concluded that DMSO concentration used in the compound library screen would not interfere with the secretion or detection of PGE2.

### High-throughput screening (HTS) of the compound library

**Figure 2:**
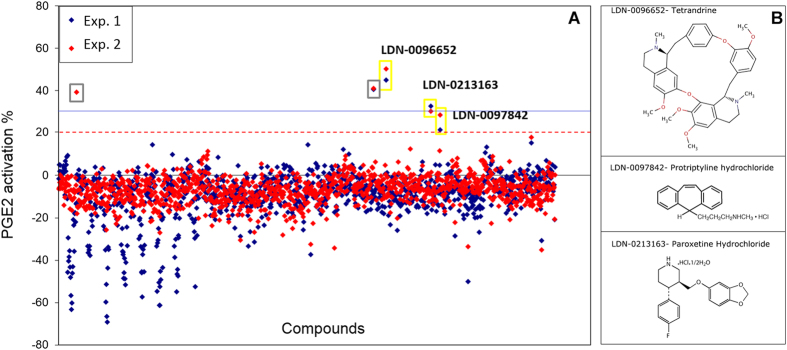
High-throughput screening with a library of known bioactive compounds identified 5 possible hits. (**A**) A total of 1402 bioactive compounds were screened in duplicate plates (blue and red dots) at a concentration of 10 μM. Following a 24-hour treatment, the activation level was presented as a ratio to unprimed cells. Five compounds were flagged as potential hits (gray and yellow boxes) and 3 were later validated (yellow boxes). (**B**) Chemical structures of these 3 compounds are shown on the right. Red dash line: positive activation threshold at 20%; blue solid line: approximate level of activation achieved by a 24-hour treatment with the positive control, 100 ng/mL TNF-α (31.5 ± 0.04%, mean + SD).

HTS of 1402 FDA-approved drugs and known bioactives was performed on MSC cultured in STEMPRO^®^ SFM at a density of 1500 cells/well. In defined conditions (Fig. 1), all compounds were screened in duplicate and at two compound concentrations, 1 μM and 10 μM. The final concentration of DMSO was 0.1%. Hits were identified based on three times the standard deviation of the basal level of activity and the activation threshold was set at 20%. While none of the compounds at 1 μM induced significant secretion of PGE2 (data not shown), 5 compounds were identified as putative PGE2 activators at a concentration of 10 μM (Fig. 2A, gray and yellow boxes). Four of the five compounds were active in both replicates.

### Hits validation and EC_50_

**Figure 3:**
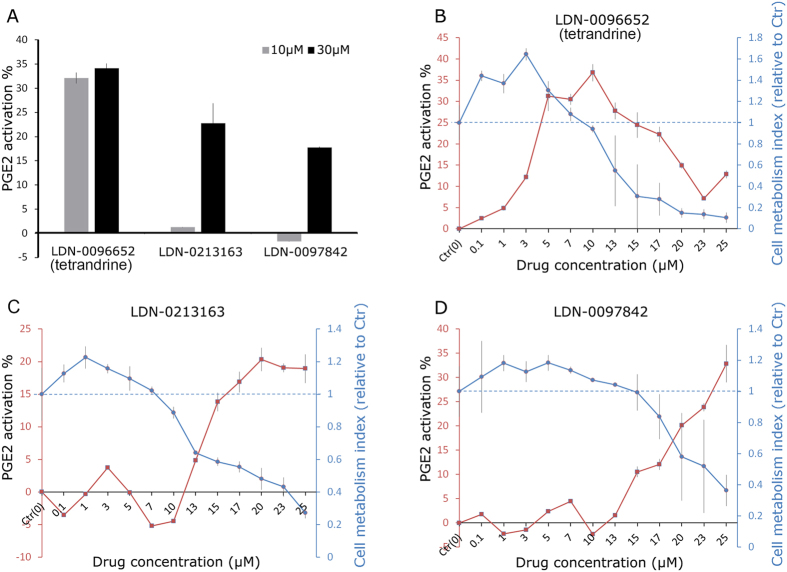
Validation of PGE2 activators and 12-point titration for EC_50_. (**A**) Each of the 3 compounds was confirmed via ELISA to activate PGE2 secretion by MSC, the activation % is presented as a ratio to unprimed cells. (**B–D**) 12-point titration for EC_50_ determination (red). The number of viable cells after 24-hour treatment (presented as metabolism index relative to unprimed cells) was measured by MTS assay (blue). Tetrandrine (LDN-0096652) was the only compound that activated PGE2 secretion without reducing cell metabolic activity. Blue dash line: metabolism index of unprimed cells.

The 5 putative PGE2 activators were assayed again in a 5-point dose response titration in quadruplicate using both HTRF and ELISA. Compound LDN-0096652 (tetrandrine), LDN-0213163 (paroxetine hydrochloride) and LDN-0097842 (protriptyline hydrochloride) were confirmed to be active in both assays (Fig. 3A showed ELISA results). Their structures are shown in Fig. 2B. To measure EC_50_ and cytotoxicity, compounds were serially diluted along a 12-point curve (0.1 μM–25 μM, Fig. 3B–D, red line). Cell metabolism index (relative to unprimed cells, 1.0 represents that compound-primed cells exhibited the same metabolic activity as unprimed cells) was measured by the MTS assay (Fig. 3B–D, blue line). All three compounds at lower concentrations showed 20–40% increased MSC metabolism over unprimed cells. Similar to the HTS result, tetrandrine at 5 μM–10 μM increased PGE2 activity by ~30–35% with minimum cell cytotoxicity. Compound paroxetine hydrochloride and protriptyline hydrochloride upregulated PGE2 secretion at 10 μM, but the effective concentrations were associated with significant cell death. We therefore chose tetrandrine as our top hit.

### Kinetics and mechanism of tetrandrine-induced PGE2 Secretion

**Figure 4:**
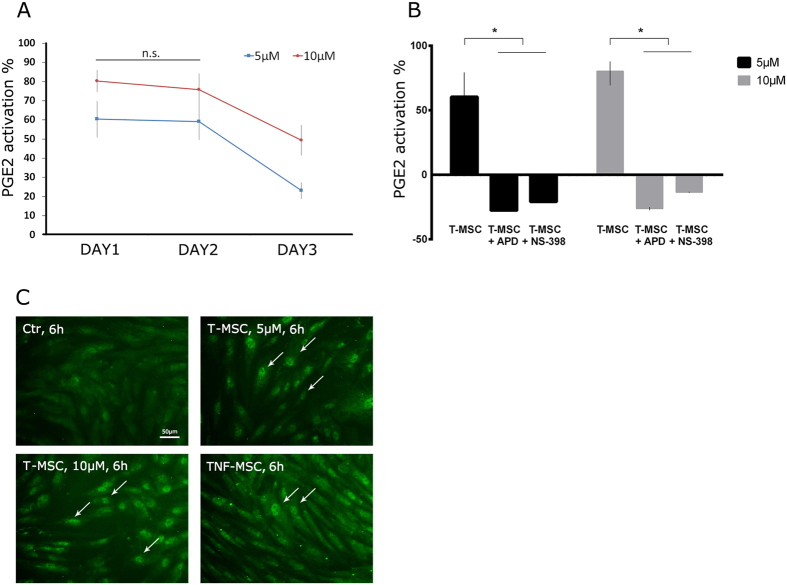
Tetrandrine activates PGE2 secretion through NF-κB/COX-2 signaling. (**A**) 1 × 10^4^ MSC were primed with tetrandrine for 24 h (DAY 1) and then rinsed and cultured in fresh medium for additional 48 h (DAY 2 and 3). On each day supernatants were collected for PGE2 quantification and fresh medium was added. After tetrandrine was removed, the activation of PGE2 secretion was sustained at the same level for up to 24 h (DAY 2) and slightly decreased on DAY 3. (**B**) Addition of NF-κB inhibitor APD or COX-2 inhibitor NS-398 together with tetrandrine for 24 h completely abolished PGE2 secretion without affecting the cell metabolic activity. (**C**) Tetrandrine and 100 ng/mL TNF-α induced nuclear translocation of NF-κB (white arrows) at 6 h. T-MSC: tetrandrine-primed MSC; *P < 0.05.

We next determined the duration of tetrandrine-induced (at 5 and 10 μM) upregulation of PGE2. Cells were treated for 24 h with tetrandrine, then tetrandrine was removed and PGE2 secretion was monitored for an additional 1 or 2 days. The activation level of PGE2 was similar after tetrandrine removal at 24 h (DAY1) (5 μM: 60.4 ± 9.5%; 10 μM: 80.3 ± 5.6%) and 48 h later (5 μM: 59.1 ± 9.5%; 10 μM: 75.8 ± 8.5%). On DAY3 the level decreased to 23.1 ± 4.2% and 49.4 ± 7.9%, respectively (P < 0.05, Fig. 4A).

As PGE2 synthesis is known to be regulated by NF-κB/COX-2 signaling^8^,^27^,^29^, we examined if tetrandrine activated the same pathway. Indeed, the upregulation of PGE2 can be entirely disrupted by the addition of 1 mM NF-κB pathway inhibitor, ammonium pyrrolidine dithiocarbamate (APD); or 5 μM COX-2 inhibitor, NS-398 (Fig. 4B), without affecting cell metabolic activity (data not shown). Interestingly, immunofluorescence staining of NF-κB revealed that tetrandrine-induced upregulation and translocation of NF-κB from cytoplasm to the nucleus within 6 h (Fig. 4C), which was later than TNF-α-induced upregulation and translocation of NF-κB that happened within 30 min (Supp. Fig. 4).

### Immunosuppression by tetrandrine-primed MSC

**Figure 5:**
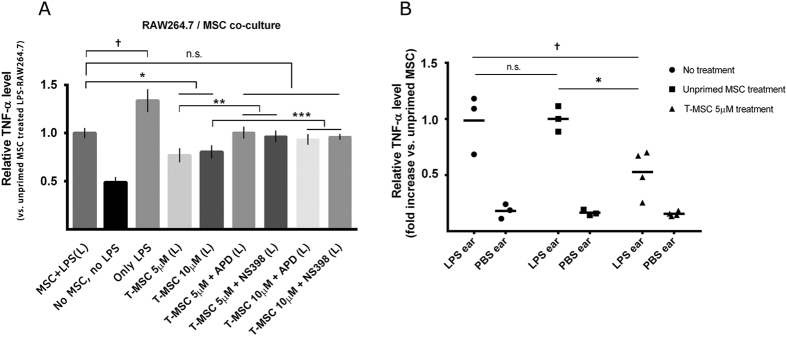
Tetrandrine-primed MSC attenuated TNF-α secretion by macrophages (*in vitro*) and in a mouse ear skin inflammation model (*in vivo*). (**A**) RAW264.7 mouse macrophages were treated with 100 ng/mL LPS for 24 h and then co-cultured with MSC primed with 5 μM or 10 μM tetrandrine for 24 h, with or without inhibitors. Mouse TNF-α in the conditioned medium was measured by ELISA. (**B**) 1 × 10^6^ tetrandrine-primed MSC were retro-orbitally injected into a mouse ear skin LPS-inflammation model. 18 h after injection, primed MSC significantly decreased the TNF-α level in the inflamed ear (LPS ear) compared to unprimed MSC, as detected via ELISA of homogenized ear tissue. T-MSC: tetrandrine-primed MSC; (L): LPS-stimulated; *, **, ***, ^†^P < 0.05.

Finally, we verified the immunosuppressive effect of tetrandrine-primed MSC both *in vitro* and *in vivo*. In MSC/RAW264.7 co-culture experiments (Fig. 5A), unprimed MSC decreased TNF-α secretion by LPS-activated RAW264.7 by ~25% (P < 0.05). Interestingly, both 5 μM and 10 μM tetrandrine-primed MSC could further attenuate the level of TNF-α (76.9 ± 3.7% and 80.4 ± 4.0% of unprimed MSC + LPS group, P < 0.05). This enhancement was completely blocked by the addition of either APD (100.6 ± 2.8%, P < 0.05) or NS-398 (96.6 ± 2.7%, P < 0.05) during MSC priming, suggesting that PGE2 and potentially other factors regulated by NF-κB/COX-2 signaling in MSC played a direct role in regulating macrophage secretion of TNF-α.

The suppression of TNF-α by tetrandrine-primed MSC was also confirmed in a mouse ear skin inflammation model (Fig. 5B). In the control group that did not receive any cell treatment, inflamed ear tissue had high amounts of TNF-α. Eighteen hours after the injection of unprimed MSC, the level of TNF-α in the inflamed ear did not significantly decrease. However, treatment with tetrandrine-primed MSC induced a significant decrease in TNF-α levels to 52.7 ± 10.3% of that in the unprimed MSC group. This indicates that tetrandrine-primed MSC, with enhanced secretion of PGE2, can achieve a much stronger immunosuppressive effect *in vivo* than that of unprimed MSC. Of note since all ear samples were immediately homogenized after harvest to preserve the half-life of TNF-α, we were not able to perform further pathological analyses in this study.

## Discussion

Using a HTS approach, we have discovered that tetrandrine can effectively upregulate PGE2 secretion of MSC at non-toxic concentrations of 5 μM and 10 μM. This response is regulated through NF-κB/COX-2 signaling and leads to enhanced immunosuppression *in vivo*.

Compared to genetic engineering of cells to promote surface expression and secretome production, priming cells with small molecules or cytokines is a potentially simpler, more cost-effective and rapid-to-perform approach. This type of transient induction to maximize therapeutic effect more closely resembles the *in situ* activation by host inflammatory mediators at pathological sites than a genetic-level modification. Priming approaches can be non-selective and selective. Non-selective strategies such as hypoxia, serum deprivation, or treatment with pleiotropic cytokines such as LPS, TNF-α, IFN-γ, activate multiple signaling pathways which collectively increase expression of downstream trophic factors or receptors^30^,^31^,^32^,^33^,^34^,^35^,^36^,^37^. Selective priming approaches target a single pathway or a limited number of related pathways to achieve a desired secretome or surface expression^38^,^39^,^40^,^41^. In this study we developed a HTS platform to identify compounds that perturb signaling pathways to enhance MSC secretion of PGE2, a potent immunosuppressive factor that regulates macrophages, T cells and dendritic cells^7^,^8^,^14^,^26^. Out of 1402 known and FDA-approved bioactive compounds, 3 compounds were validated by both HTRF and ELISA assays, namely tetrandrine, paroxetine hydrochloride, and protriptyline hydrochloride. Paroxetine hydrochloride and protriptyline hydrochloride displayed high cytotoxicity at active concentrations. Only tetrandrine can activate MSCs at 5 μM and 10 μM with minimum cytotoxicity observed.

The immunoregulatory activity of MSC is at least in part achieved via the secretion of a variety of immunosuppressive factors, such as PGE2, IL-10, TGF-β, nitric oxide, TNF-α-induced protein 6 (TSG-6), and IDO^6^,^7^,^8^,^11^,^12^,^27^,^29^. In our earlier work, we transduced MSC with IL-10 and homing ligands to enhance targeting to inflamed tissues, and observed reduced inflammation in a mouse ear inflammation model^20^. Several studies have postulated PGE2 as one of the primary soluble mediators of immunomodulatory function in MSC^11^,^12^,^27^,^28^,^29^. PGE2 secreted by MSC can induce the conversion of macrophages from a pro-inflammatory (M1) to anti-inflammatory phenotype (M2)^8^,^14^. Inhibition of PGE2 also has been shown to significantly mitigate MSC-mediated immunosuppression on both dendritic cells and activated T cells^7^,^26^. A recent study showed that the immunomodulatory ability of hMSC gradually declines with consecutive passages due to the alteration of COX-2 and PGE2 levels^42^.

Tetrandrine (CAS No. 518-34-3) is a bis-benzyl-isoquinoline alkaloid originally isolated from a Chinese medicinal herb, *Radix Stephanae tetrandrae*. Tetrandrine has been used traditionally to treat congestive circulatory disorders and has been reported to modulate pathways associated with Ca^2+^ regulation, affecting endothelium-dependent relaxation, apoptosis, angiogenesis, anti-oxidation, and anti-inflammation^43^. It has been shown to inhibit voltage-gated Ca^2+^ channels (IC_50_ = 10.1 μM), type II maxi-Ca^2+^-activated K^+^ channels (IC_50_ = 0.21 μM) of rat neurohypophysial terminals^44^, and Ca^2+^-activated chloride channel in cultured human umbilical vein endothelial cells (IC_50_ = 5.2 μM)^45^. Other studies have utilized tetrandrine at non-cytotoxic concentrations ranging from 0.1 μM to 5 μM, depending on cell type^46^,^47^,^48^. In this study, we verified that tetrandrine concentrations below 10 μM were non-toxic to MSC since cell mitochondrial/metabolic activity and morphology were not significantly affected. While this study focused on tetrandrine, its molecular structure can potentially serve as a reference for further screening and optimization of small molecules that activate PGE2 secretion.

The activation and translocation of NF-κB is involved in regulating the transcriptional activation of specific target genes and the expression of several pro-inflammatory factors^49^,^50^. When activated, NF-κB separates from its inhibitor IκB and translocates from the cytoplasm to the nucleus where it controls gene expression, particularly activating COX-2 which catalyzes PGE2 synthesis^51^. Tetrandrine has been reported to be associated with NF-κB signaling. Dang *et al*. recently showed that tetrandrine at 0.1 μM to 1 μM can suppress the NF-κB and ERK signaling pathways in LPS-activated microglia^47^. Specifically, at 50 μM, tetrandrine abolished binding of NF-κB to DNA and inhibited TNF-α release from LPS-activated rat primary microglial cells^48^ and at 5 μM, tetrandrine can downregulate binding of NF-κB to DNA when used to prime T cells before activating the T cells with inflammatory stimuli^46^. In contrast to the reported inhibitory effect of tetrandrine on NF-κB, our study suggests that with MSC, NF-κB activation is associated with tetrandrine-induced upregulation of PGE2. This is evidenced by data showing that (i) the NF-κB inhibitor APD or the COX-2 inhibitor NS-398 blocked PGE2 secretion by MSC entirely, and (ii) NF-κB translocation was significantly more pronounced when tetrandrine or TNF-α was added to MSC. Moreover, tetrandrine-induced NF-κB translocation was observed only after 6 h of incubation, which is relatively slow since TNF-α triggered NF-κB translocation within 30 min. Nevertheless, the activation could be sustained up to 48 h after tetrandrine was removed from culture. Our data suggest that despite the NF-κB suppression effect on cell types such as microglial and T cells, tetrandrine can trigger the activation of the NF-κB signaling in MSC. Finally, it is possible that immunomodulatory factors regulators other than PGE2 were upregulated through NF-κB or other pathways during tetrandrine treatment and additional studies are required to examine this.

Three observations in this study should be noted. First, the magnitude of enhancement of PGE2 secretion from MSC depended on whether MSC were treated in a 384- or 96-well format. In 384-well-format (1500 cells/well, 30 cells/μL), the average activation level of PGE2 by tetrandrine was ~30%, while in 96-well-format with similar dose per cell (5000 cells/well, 25 cells/μL) it reached over 60%. This trend was repeatedly observed in this study. Secondly, MSC cultured in STEMPRO^®^ MSC SFM before activation exhibited much lower but more consistent expression (<50 pg/mL per well with seeding density at 1500 cells/well, coefficient of variation <5%) of PGE2 compared to cells cultured in serum-containing αMEM (~200 pg/mL per well per well with seeding density at 800 cells/well, data not shown). Similarly, another study reported lower PGE2 secretion when MSC were cultured in serum-free media compared to serum-containing media^37^. Thirdly, it is possible that incubation with tetrandrine at lower concentration, i.e. ≤7 μM, may stimulate MSC metabolism as indicated in Fig. 3B. Therefore the activation of PGE2 by 5 μM tetrandrine may be amplified by the increased metabolic activity. Meanwhile, we used MTS assay to measure the mitochondrial/metabolic activity, which might not reflect the true number of cells. The correlation between tetrandrine-induced cell metabolism and PGE2 activation, as well as the difference between PGE2/metabolic activity and PGE2/cell numbers require further investigation. Of note, tetrandrine treatment for 24 h did not alter the expression of MSC surface markers including CD73, CD90, CD44, CD105, CD34, CD11b, CD45, CXCR4, CD166, CD162, CD106, CD104, CD102, CD54, CD49d, CD49a, CD29, CD15, CD11a.

Maggini J, *et al*. reported that PGE2 produced by un-primed MSC was able to inhibit the production of TNF- α by activated macrophage^52^. In this study, the immunosuppression effect by MSC was enhanced following tetrandrine activation. When co-cultured with LPS-stimulated RAW264.7 macrophages, tetrandrine-primed MSC significantly attenuated TNF-α secretion by the macrophages, compared to unprimed MSC. Similar to previous reports^53^, we did not detect altered expression of CD206 on RAW264.7 in response to PGE2 (data not shown). Furthermore, in an LPS-induced mouse ear skin inflammation model, only the group that received tetrandrine-primed MSC showed a decreased level of TNF-α in the inflamed ear. The infusion of unprimed MSC did not exhibit any effect. This is similar to previous reports where local expression of TNF-α, IL-1β and IL-10 remained unchanged after implantation of unprimed MSC^54^. Interestingly, we have observed a similar trend that was reported in a recent study. By injecting MSC primed with a small molecule that can enhance the homing ability, we can reduce the TNF-α level in inflamed ear to ~50% of the level for unprimed MSC treatment and effectively decrease the inflamed ear thickness^55^. It has been suggested that the immunosuppressive effect of PGE2 requires MSC-to-macrophage contact^8^. For instance, PGE2 secreted by MSC can signal adjacent macrophages through the EP4 receptors and convert them to an anti-inflammatory phenotype^14^. However, the window for such cell-cell contact is limited *in vivo* since MSC largely depend on a “hit-and-run” mechanism^20^,^56^. We have shown that retro-orbitally injected MSC begin to extravasate at the site of inflammation as early as 2 h and almost 50% of the MSC complete extravasation within 6 h^57^. In this study, enhanced PGE2 secretion was sustained for 48 h after tetrandrine was removed from culture. We anticipate that in the initial 24–48 h following transplantation, tetrandrine-primed MSC, compared to unprimed MSC, can more efficiently suppress local macrophages at sites of inflammation by transiently secreting much more PGE2. Moreover, Pelus LM and Hoggatt J. reported that PGE2 can indeed enhance hematopoietic stem cell (HSC) engraftment by enhancing stem cell homing, survival and self-renewal^58^,^59^. Short-term exposure of HSC to PGE2 increases CXCR4 receptor expression. Durand EM, *et al*. also reported that an *in-vivo* interaction between PGE2 and the Wnt signaling pathways controls HSC engraftment^60^ Since 50% of the transplanted MSC engrafted within 6 h and the transient activation of PGE2 can last for more than 24 h post injection, it is reasonable to speculate that tetrandrine activation might also result in higher cell engraftment and contribute to enhanced immunosuppression. Future studies are required to unveil the underlying mechanism.

The enhancement of PGE2 secretion can effect a variety of other factors besides TNF-α. Indeed, cytokines like PGE2, IDO, IL-4, IL-10, and TGF-ß are immunoregulatory and their expression can be simultaneously upregulated during inflammation through interactive signal pathways^61^. It is known that PGE2 can upregulate IDO1 expression in circulating DCs and induce immune tolerance^62^. Injection of PGE2 can enhance IL-10 and attenuate TGF- ß1 expression in aged rats^63^. The interplay between PGE2/COX2 and TGF- ß has been revealed by other studies^64^,^65^. It will be of interest to understand whether over expression of PGE2 by MSC can modulate other anti-inflammatory factors secreted by DCs, T cells and macrophages such as IL-10 and IDO both *in vitro* and *in vivo*.

Our current work establishes a framework for subsequent, more sophisticated HTS assays such as multiplexing readouts of several immunoregulatory factors including IL-6, TSG-6, NO, IDO, IL-1, and IL-10. It is important to note that the HTS was performed on bone marrow derived MSC and whether the effect of tetrandrine is conserved across different sources of MSC (e.g. adipose vs. bone marrow derived) or across multiple MSC donors requires further investigation. Given that adipose derived MSC express different surface markers^66^ and have higher proliferative capacity^67^, MSC from adipose tissue may respond differently to tetradrine priming. Also, tetrandrine may additionally regulate immune properties of MSC other than PGE2 secretion. The exact signaling pathways underlying tetradrine/PGE2 activation, e.g. the classical or the alternative pathway to activate NF-κB, as well as the molecular targets through which tetradrine mediates the immune properties of MSC remain to be investigated. Additionally, more functional data is required to support the hypothesis that a small molecule preconditioning regimen can enhance the therapeutic effect.

The potential utility of a small molecule-based approach to enhance MSC immunosuppression could be far-reaching. For instance, in addition to a simple pre-conditioning regimen, we have previously demonstrated a “particle-in-cell” platform that enables storage and controlled release of small molecules from MSCs for weeks^22^,^68^,^69^. This platform enables small molecules such as tetrandrine to continually stimulate transplanted cells for weeks as the particles degrade. Identified small molecules can also be immobilized within implantable cell-encapsulation devices or scaffolds to controllably stimulate transplanted cells. In conclusion, high-throughput screening of small molecules for targeted cell activation is a versatile strategy to identify candidates that specifically stimulate important cell biological pathways and to enhance the therapeutic potential of transplanted cells. This technology may also find utility in other immune disease models such as inflammatory bowel disease, colitis and sepsis^70^,^71^, where pathological changes could be mediated by small molecules such as PGE2.

## Methods

### Cell and culture conditions

All methods regarding human subjects were approved by the Harvard University Committee On the Use of Human Subjects. All experimental methods were carried out in accordance with the approved protocols. Bone-marrow derived primary human MSC from two healthy donors were obtained from the Texas A&M Health Science Center, Institute for Regenerative Medicine (Temple, TX). Informed consent was obtained from all subjects. MSC at passage 3–5 were cultured in either serum-containing or serum-free medium. The serum-containing medium comprised αMEM (Life Technologies, Carlsbad, CA) supplemented with 10% FBS (Atlanta Biologicals, Lawrenceville, GA), 1% L-Glutamine (Life Technologies, Carlsbad, CA) and 1% Penicillin-Streptomycin (Life Technologies, Carlsbad, CA). The serum-free medium consisted of STEMPRO^®^ MSC SFM (Life Technologies, Carlsbad, CA) supplemented with 1% Penicillin-Streptomycin and 1% L-Glutamine. The mouse leukemic monocyte macrophage cell line RAW264.7 (ATCC, Manassas, VA) was used to verify the immunosuppressive effect of MSC^14^. RAW264.7 was cultured in αMEM supplemented with 10% FBS, 1% L-Glutamine and 1% Penicillin-Streptomycin.

### High-throughput screening (HTS) assay

MSC were treated in a 384-well plate with a library containing 1402 known and FDA-approved bioactive compounds (Prestwick, France; MicroSource, CT, USA). Figure 1 illustrates the 4-day HTS workflow. On day 1, cells were seeded into 384-well plates at a density of 1500 cells/well using Thermofisher Multidrop Combi (Thermo Scientific, Waltham, MA) and incubated for 24 h. Compounds were then transferred to the cell plates on day 2 for a final compound concentration of 1 or 10 μM in 0.1% DMSO via Beckman Coulter Biomek NX Automated Liquid Handling System (Beckman Coulter, Brea, CA) and incubated for 24 h. On day 3, 10 μL of supernatant was transferred to a white 384-well low-volume microplate (Proxiplate, PerkinElmer, Waltham, MA). The level of secreted PGE2 was detected by using Homogeneous Time-Resolved Fluorescence (HTRF) technology, a combination of fluorescence resonance energy transfer technology (FRET) with time-resolved measurement (62P2APE, Cisbio, Bedford, MA). The number of viable cells (indicated by metabolic activity) after compound treatment was detected by performing formazan production-based MTS cell metabolic activity assay (CellTiter 96^®^ AQueous One Solution Cell Proliferation Assay MTS, Promega, Madison, WI) for 2 h. On day 4, the HTRF assay plates were read via a PerkinElmer EnVision Multilabel Plate Reader (PerkinElmer, Waltham, MA). The percentage of activation was calculated using the following formula:


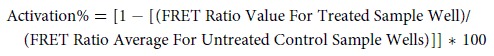


### Validation of hits

Confirmation of the hits was performed in a 5-point dose response titration via both HTRF and ELISA (Prostaglandin E2 Parameter Assay Kit, R&D Systems, Minneapolis, MN) assays. Validated hits demonstrated activity from two different assay formats and reagents thereby reducing the chance that they were selected based on artifacts of detection. The EC_50_ of compounds was determined from a 12-point dose response titration via HTRF assay.

### Signaling pathway identification

MSC were seeded at 1 × 10^4^ cells/well in 96-well plates. One day later cells were treated with 5 μM or 10 μM tetrandrine, with or without 1 mM ammonium pyrrolidine dithiocarbamate (APD) (Sigma-Aldrich, St. Louis, MO) or 5 μM NS-398 (Cayman Chemical, Ann Arbor, MI), which selectively inhibit NF-κB and COX-2 respectively. After 24 h, supernatants were collected and assayed for PGE2 via HTRF assay. In another set of experiments, MSC were seeded in 24-well plates at a density of 3 × 10^4^ cells/well for 24 h and treated with 5 μM or 10 μM tetrandrine for 30 min or 6 h. Cells were then fixed with 4% paraformaldehyde, permeabilized with 0.1% Triton-X, and incubated with rabbit anti-human NF-κB/p65 antibody (622602, Biolegend, San Diego, CA) and FITC-conjugated goat anti-rabbit antibody (F9887, Sigma-Aldrich, St. Louis, MO). Nuclear translocation of NF-κB was visualized using an epifluorescence microscope (Nikon Eclipse TE2000-U, Japan).

### Kinetics of tetrandrine-induced upregulation of PGE2

MSC were seeded at 5000 cells/well in 96-well plates for 24 h and then treated with 5 μM or 10 μM tetrandrine. Following an additional 24 h, supernatants (Day 1) were collected and replaced with fresh medium every 24 h until Day 3. The amount of PGE2 secreted every 24 h was detected via HTRF assay.

### Immunosuppressive effect of tetrandrine-primed MSC *in vitro*

RAW264.7 cells were seeded at 2 × 10^4^ cells/well in 96-well plates for 24 h and activated with 100 ng/mL lipopolysaccharides (LPS) from *Salmonella enterica* serotype *typhimurium* (Sigma-Aldrich, St. Louis, MO) for an additional 24 h. MSC unprimed or primed with 5 μM or 10 μM tetrandrine for 24 h were seeded at 1 × 10^4^ cells/well directly on top of RAW264.7 cells. Supernatants were collected 24 h later. Mouse TNF-α in the supernatants was quantified using ELISA (Biolegend, San Diego, CA).

### Animal study

All animal experimental protocols were reviewed and approved by the MGH Subcommittee on Research Animal Care. All experimental methods were carried out in accordance with the approved protocols. We used a mouse ear skin inflammation model to test the *in vivo* therapeutic effect of tetrandrine-primed MSC. Ten C57BL/6 mice were anesthetized by intramuscular injection of a combination of anesthetics (80 mg/kg ketamine and 12 mg/kg xylazine). Lipopolysaccharide (LPS) (30 μg in 30 μL saline) was injected intradermally into the dorsal side of the left ear using an insulin syringe. The right ear was injected with 30 μL saline as an internal control. After 24 h, animals were divided into three groups: control group (n = 3) received no cell treatment; unprimed MSC group (n = 3) received 1 × 10^6^ unprimed cells; tetrandrine-primed MSC (T-MSC) group (n = 4) received 1 × 10^6^ cells primed with 5 μM tetrandrine. All cells were injected retro-orbitally. At 18 h after injection, mice were sacrificed and both ears were harvested. The tissue samples were then homogenized in ice-cold extraction buffer (RIPA with 0.5% Tween-20) and homogenates were transferred to 1.5-mL microcentrifuge tubes followed by centrifugation at 13,000 × g for 10 min at 4 °C. The supernatants were stored at −80 °C until analysis. The levels of mouse TNF-α in the samples were quantified using an anti-mouse TNF-α ELISA kit (Biolegend, San Diego, CA).

### Statistical analysis

Unless otherwise stated, experiments were performed at least in triplicate, and data are presented as mean ± standard error of the mean. Unpaired Student’s t-test and one-way ANOVA with Scheffe’s test for posthoc comparison were used to compare group means, after testing for normality and equal variance of the data.

All statistical analyses were carried out in Graphpad Prism^®^. Statistical significance was inferred at a 2-sided *p* ≤ 0.05.

## Additional Information

**How to cite this article**: Yang, Z. *et al*. Tetrandrine identified in a small molecule screen to activate mesenchymal stem cells for enhanced immunomodulation. *Sci. Rep.* **6**, 30263; doi: 10.1038/srep30263 (2016).

## Supplementary Material

Supplementary Information
