# Supplementary material for: Tetrandrine identified in a small molecule screen to activate mesenchymal stem cells for enhanced immunomodulation
Source: Sci Rep. 2016 Jul 26;6:30263. doi: 10.1038/srep30263 (PMC4960598; doi:10.1038/srep30263)

Tetrandrine identified in a small molecule screen to activate mesenchymal stem cells for enhanced immunomodulation

Zijiang Yang[1,2,4,5,7], John Concannon[3], Kelvin S. Ng[1,2,4], Kathleen Seyb[3], Luke J. Mortensen[8], Sudhir Ranganath[1,2,4,9], Fangqi Gu[1,2,4], Oren Levy[1,2,4], Zhixiang Tong[1,2,4], Keir Martyn[1,2,4], Weian Zhao[6], Charles P. Lin[2,5], Marcie A. Glicksman[2,3,], Jeffrey M. Karp[1,2,4,*]*

[1]Harvard-MIT Health Sciences and Technology; [2]Harvard Medical School; [3]Laboratory for Drug Discovery in Neurodegeneration, Harvard NeuroDiscovery Center, Brigham and Women's Hospital; [4]Brigham and Women's Hospital, Harvard Stem Cell Institute; [5]Wellman Center for Photomedicine, Massachusetts General Hospital; [6]Department of Pharmaceutical Sciences, Sue and Bill Gross Stem Cell Research Center and Chao Family Comprehensive Cancer Center, Department of Biomedical Engineering, and Edwards Lifesciences Center for Advanced Cardiovascular Technology, University of California; [7]Advanced Industrial Technology Research Institute, Shanghai Jiao Tong University, Shanghai, China; [8]Regenerative Bioscience Center, Department of Animal and Dairy Science, and College of Engineering, University of Georgia; [9]Department of Chemical Engineering, Siddaganga Institute of Technology, Tumkur 572103, India.

*denotes co-corresponding authors. Correspondence may be directed to JMK (65 Landsdowne St, Cambridge, MA 02139; http://www.karplab.net; +1 (617) 817-9174; jeffkarp.bwh@gmail.com) or MG (65 Landsdowne St, Cambridge, MA 02139; +1 (617) 768-8661; mglicksman@rics.bwh.harvard.edu)

Supplementary Figure Legends

Supp. Fig. 1. TNF-α and IFN-γ activate PGE2 secretion by MSC. (A) MSC were incubated in a 384-well plate with 100 ng/mL TNF-α or 100 ng/mL IFN-γ for 6, 12 and 24 hours. **A1**: absolute PGE2 concentrations determined by HTRF; **A2**: activation level, presented as the percentage increase of PGE2 secretion by treated cells compared to untreated cells at each time point. **(B)** 24-hour treatment with TNF-α or IFN-γ did not induce cytotoxicity compared to untreated control (Ctr). Seeding density was 800 cells/well in αMEM, 50 μl/well. Cell mitochondrial/metabolic activity was determined using MTS assay. *, † : $P < 0.05$.

Supp. Fig. 2. MSC cultured in serum-free media exhibited distinct morphology and sensitivity. (A) MSC were smaller and more spindle-shaped when cultured in serum-free STEMPRO® SFM than in serum-containing αMEM. **(B)** MSC cultured in STEMPRO® SFM were significantly more reactive to 100 ng/mL TNF-α in PGE2 secretion. *: $P < 0.05$.

Supp. Fig. 3. Development of HTS protocol in serum-free media. (A) 0%, 0.5% or 2% DMSO did not affect the ability of HTRF to detect 250 pg/mL recombinant PGE2 after a 24-hour incubation. **(B)** DMSO up to 2% for 24 hours did not affect viability of MSC cultured in serum-free medium. **(C)** Enhancement of PGE2 secretion by TNF-α was intact at 0.5% DMSO but diminished at 2% DMSO. *: $P < 0.05$.

Supp. Fig. 4. Tetrandrine treatment for 30 min did not induce activation and translocation of NF-κB from cytoplasm to nucleus. Incubation of 30 min is sufficient for 100 ng/mL TNF-α but insufficient for 5 or 10 μM tetrandrine to induce nuclear translocation of NF-κB (white arrows). T-MSC: tetrandrine-primed MSC.

Supp. Fig. 1

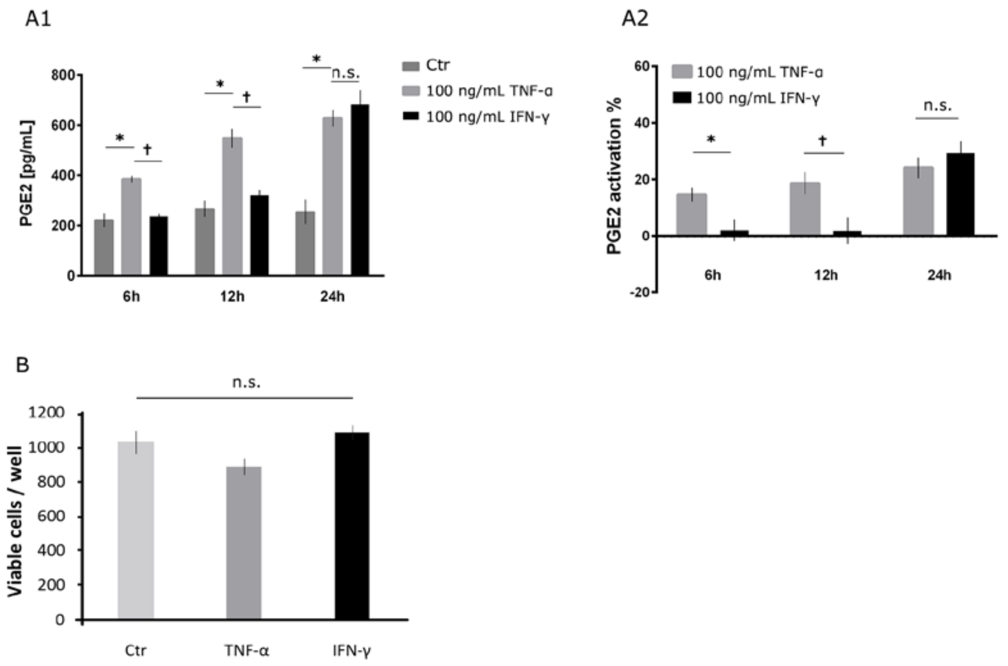

Supp. Fig. 2

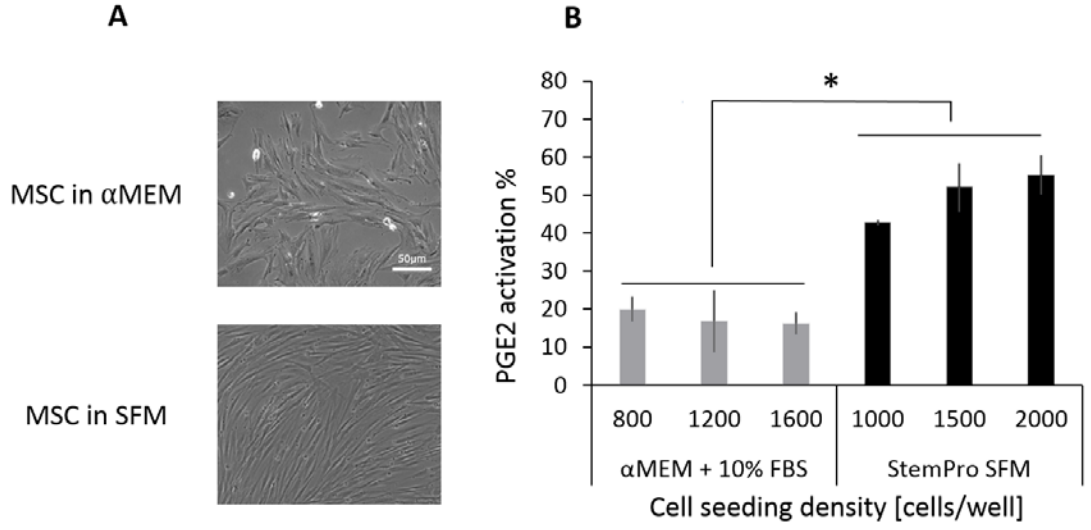

Supp. Fig. 3

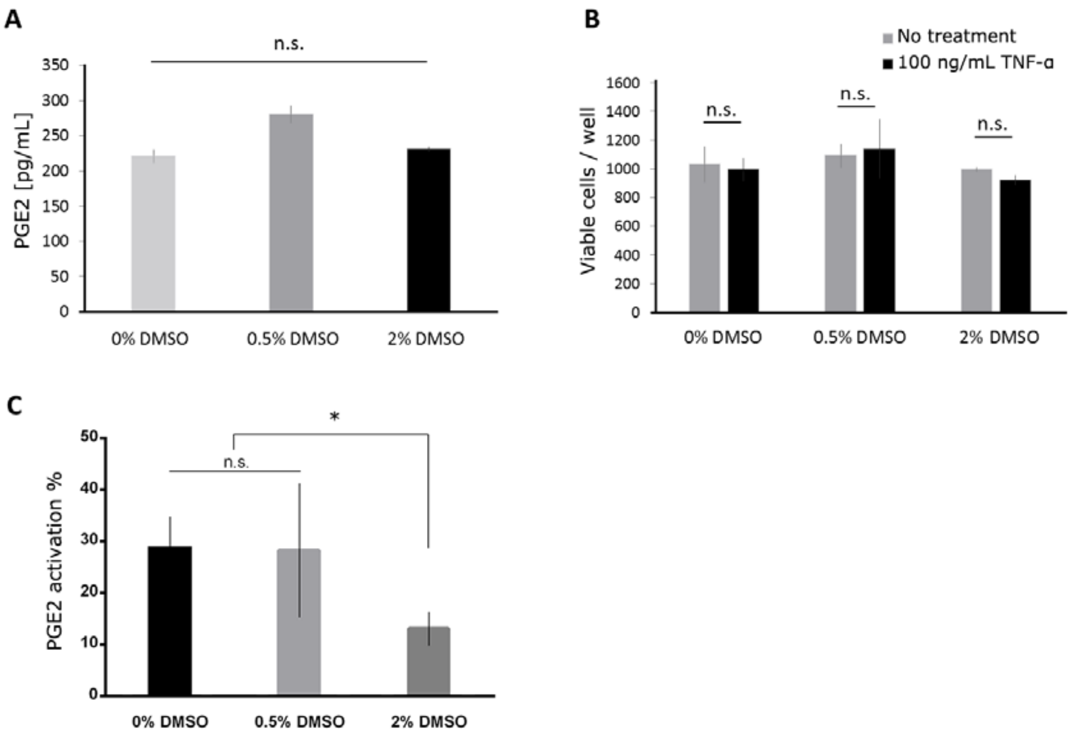

Supp. Fig. 4

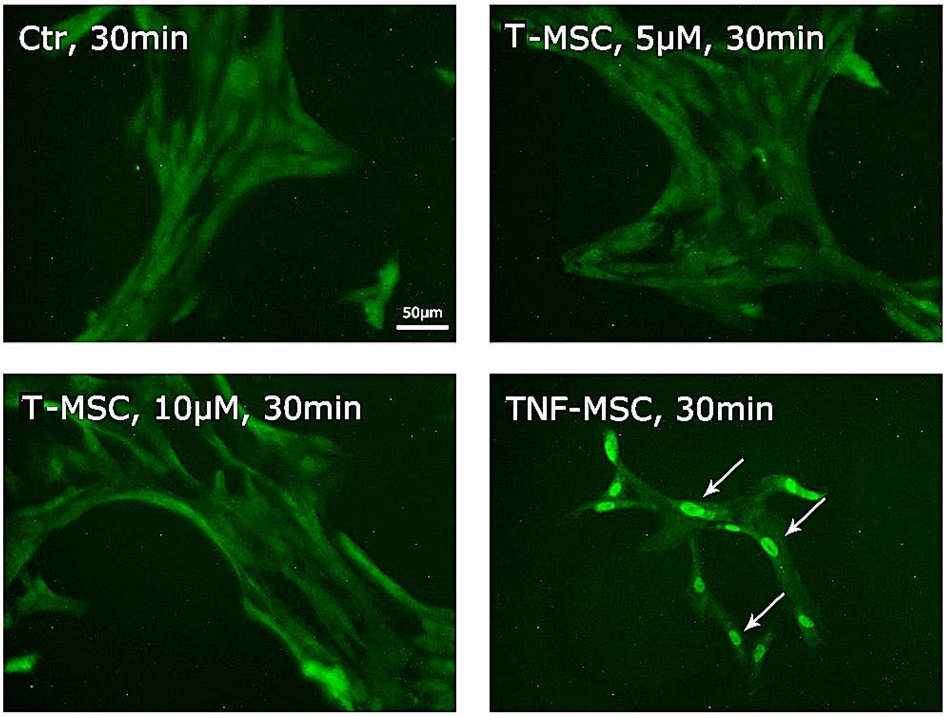

Supplement: Supplementary Information [file srep30263-s1.pdf]
